# Long-Term Vector Integration Site Analysis Following Retroviral Mediated Gene Transfer to Hematopoietic Stem Cells for the Treatment of HIV Infection

**DOI:** 10.1371/journal.pone.0004211

**Published:** 2009-01-16

**Authors:** Jun Hayakawa, Kareem Washington, Naoya Uchida, Oswald Phang, Elizabeth M. Kang, Matthew M. Hsieh, John F. Tisdale

**Affiliations:** Molecular and Clinical Hematology Branch (MCHB), National Institutes of Diabetes and Digestive and Kidney Disorders (NIDDK) and National Heart, Lung, and Blood Institute (NHLBI), National Institutes of Health (NIH), Bethesda, Maryland, United States of America; University of Florida, United States of America

## Abstract

We previously reported the efficacy of nonmyeloablative allogeneic transplantation in 2 HIV positive recipients, one of whom received retrovirus transduced hematopoietic stem cells to confer resistance to HIV. Here we report an assessment of retroviral integration sites (RISs) recovered out to 3 years post-transplantation. We identified 213 unique RISs from the patient's peripheral blood samples by linear amplification-mediated PCR (LAM-PCR). While vector integration patterns were similar to that previously reported, only 3.76% of RISs were common among early (up to 3 months) and late samples (beyond 1 year). Additionally, common integration sites were enriched among late samples (14.9% vs. 36.8%, respectively). Three RISs were found near or within known oncogenes, but 2 were limited to early timepoints. Interestingly, an integration site near the MDS1 gene was detected in long-term follow-up samples; however, the overall contribution of MDS1 integrated clone remained stably low during follow-up.

## Introduction

The establishment of safe and effective vector delivery systems for gene therapy applications appears achievable; however, the adverse events reported from recent gene therapy trials have lead to a reassessment of the risks associated with vector insertional mutagenesis [1], [2]. In the first such report, a clonal lymphoproliferation was induced by murine leukemia virus (MLV) vector mediated insertional activation of the *LMO2* gene in patients treated for severe combined immunodeficiency [3]. The potential for such clonal expansion has recently been described even in the context of a disorder for which no known advantage for genetically corrected cells exists. The use of the SFFV long terminal repeat (LTR), which expresses well in myeloid cells, may have driven the expansion of *MDS1-EVI1*, *PRDM16*, or *SETBP1* integration containing myeloid cells observed in patients treated for chronic granulomatous disease using a similar approach [4], [5]. Hence, the elucidation of vector insertion mediated oncogenesis is important to ascertain the risk of gene therapy approaches utilizing integrating vectors. Linear amplification-mediated PCR (LAM-PCR) was initially introduced for detecting and sequencing unknown DNA flanking sequences at vector integration sites to track stem cell clones' contribution to in vivo hematopoiesis [6], [7]. A number of investigators have now demonstrated that murine leukemia virus (MLV) vectors have a propensity to integrate around transcription start sites (TSS) [8] with a higher than expected integration frequency within 2 introns of *Mds1/Evi1* gene [5], which was previously identified as a component of *MDS-EVI1* translocation 3∶21 found in human acute myelogenous leukemia (AML) [9]. These data have prompted some to propose limiting both vector copy number as well as transduced stem cell dose to reduce the risk of insertional mutagenesis [10]. Further data, especially long term analyses generated from human clinical trials, are required to assess the risk of integrating vectors. Additionally, previous integration site analyses from human clinical trials did not focus on the integration patterns from short term (ST-HSCs) versus long-term hematopoietic repopulating cells (LT-HSCs). To investigate these questions, we performed exhaustive LAM-PCR on patient samples from a phase I/II clinical trial for gene therapy of HIV infection.

## Results

**Figure 1:**
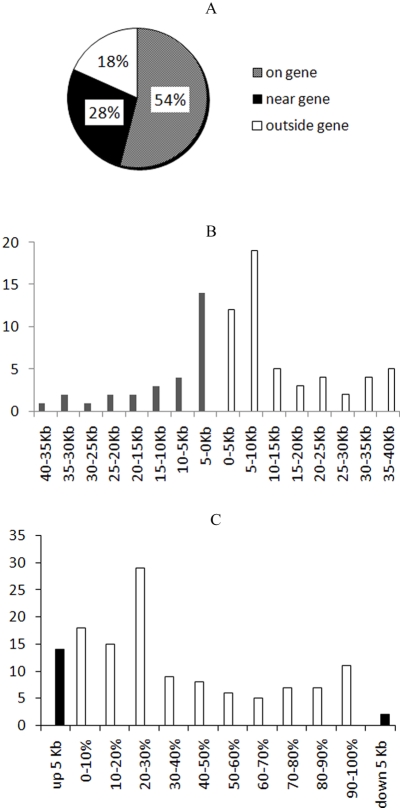
Figure 1A. Pie chart of retroviral integration sites (RISs) from 213 unique sites. Percents are shown; near gene is defined as 100 Kb up- or downstream of transcription units. Figure 1B. Genomic distribution of RISs with respect to the distance from transcription start sites (TSSs). The frequency (%) of RISs is plotted versus 40 kilobases (Kb) upstream (gray bars) or downstream (white bars) of TSSs. Figure 1C. RISs in and near gene coding regions. RIS locations inside genes are expressed as the percentage of the overall length of each individual vector targeted gene. Upstream 5 kilobases (up 5 Kb) or downstream 5 Kb (down 5 Kb) are denoted in black bars. All other RISs are located within 5 Kb of TSS.

TasI enzymes and TaiI enzymes were used (Supplemental Table S1) during LAM PCR, with a switch to TaiI to avoid sampling artifact and to circumvent the frequent internal vector sequences obtained using the TasI enzyme. After optimization, over 1000 sequences were analyzed from which 213 unique integrations were obtained. There were no new RISs were identified in the last 300 sequenced samples, indicating there was adequate integration sampling. Both TasI and TaiI enzymes identified overlapping RISs, but the vector internal control sequences were significantly reduced when we used TaiI enzymes. 213 unique RISs were isolated from the patient's peripheral blood myeloid and lymphoid cells from 1 to 36 months after reinfusion of genetically modified CD34+ cells (Supplemental Tables S2 and S3). Figure 1A shows the location of vector integrants with respect to known genes (RefSeq genes). Overall, 54% of detected RISs were located within genes and 28% were within 100 kb from the start or end of genes. Figure 1B illustrates the relative distribution of RIS around the TSS; 49% of the integrations occurred within 10 Kb up- or downstream of TSSs. When considering the entire length of a targeted RefSeq gene among RISs found within genes, the RISs were found predominantly within the first 30% of the total gene length from the TSS (Figure 1C).

**Figure 2:**
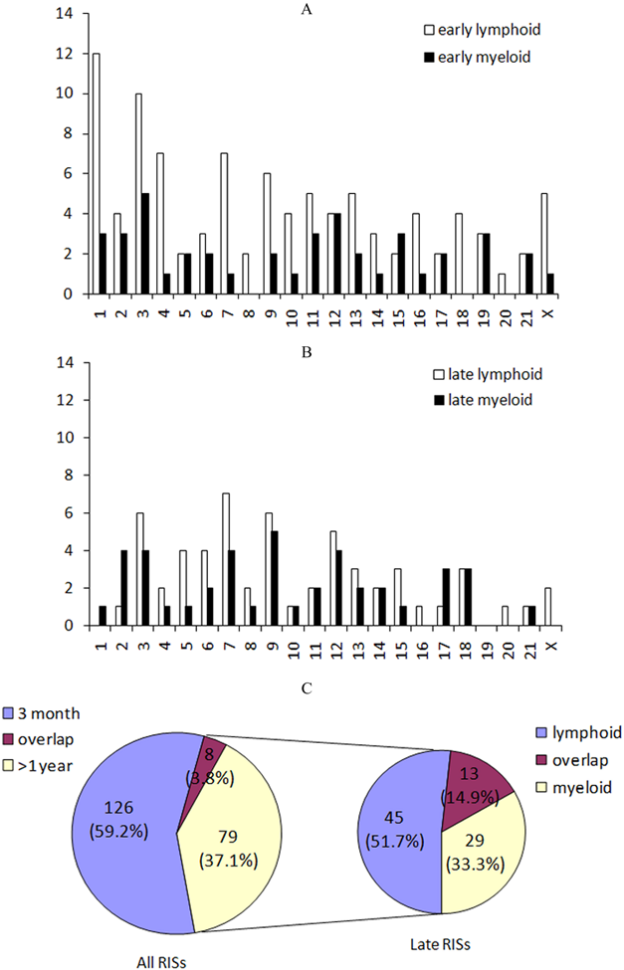
Distribution of RISs. 2A shows the RISs from the samples up to 3 months post transplant (early); 2B contains samples over 1 year post transplant (late). Lymphoid (white bars) and myeloid (black bars) are distinct each other. X-axis refers to the chromosome number; Y-axis, the number of RIS. 2C depicts the overlap of RIS between early stage and late stage. Among all RISs, there were only 8 of 213 overlapping between early and late samples, equivalent to 3.76%. Only 13 of 87 RISs were overlapping between lymphoid and myeloid from the late samples, equivalent to 14.9%.

Figures 2A & B show the chromosomal distribution pattern of RISs between lymphoid and myeloid cells from early or late post transplant. As expected, gene dense chromosomes, e.g. 1, 3, 7, and 9, contained more RISs. The pattern of chromosomal integration appeared different between early and late RISs. There were 134 integrations up to 3 months, and 87 integrations beyond 1 year. Among the late (>1 year) samples, 58 integrations were among lymphoid and 42 among myeloid progeny. Interestingly, only 3.76% (8 of 213) of RISs were shared between early and late phase samples (Figure 2C), and 14.9% (13 of 87) between lymphoid and myeloid populations at time points 1 year or greater post transplant (Figure 2C). This pattern is somewhat surprising as cells derived from LT-HSCs were previously thought to originate from a shared pool of genetically modified cells.

**Figure 3:**
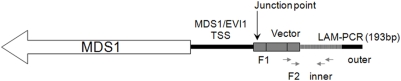
Location of the RIS identified upstream of the MDS1/EVI1 TSS. The black arrowed indicate the insertion site at chromosome 3(q26.2) position 170865174 which is 1006 bp upstream of the TSS. The black vertical arrow is junction point of our vector to TSS (position 170865174). The vertical stripes section shows the 193 bp where our sequence was detected by LAM-PCR. The gray arrows are vector specific PCR primers (F1-outer, F2-inner) to confirm the insertion.

**Figure 4:**
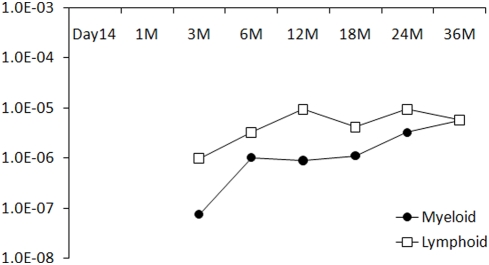
QPCR by Taqman probe measuring the contribution of RIS in near the MDS1/EVI1 TSS. X-axis denotes time points after transplantation; M, month(s). Y-axis denotes the % of level of integrated vector. Up to 3 months, the clone was undetectable.

Three RISs were found on or near the TSS of known oncogenes. It has been estimated that the probability of activating a given gene from a single integration event is >10^−5^ [11]. Insertions in integrin alpha 9 (*ITGA9*), located in a region of frequent homozygous deletions in tumor samples [12], and ADP-ribosylation factor-like 11 (*ARL11*), a genetic variant of which predisposes to familial cancer[13], were detected in the early phase, but never among the late phase samples. With regard to the late phase samples, one RIS was found 1006 base pairs upstream of *MDS1* (Figure 3), which was reported to be involved in chromosomal translocations in human myeloid leukemias [9] and frequently found as a common RISs in MLV vector system [4], [5], [8]. Other previously identified CISs: *LMO2*, *PRDM16*, as well as *SETBP1*, which are involved in uncontrolled proliferation, abnormal hematopoiesis, and leukemogenesis [4], were not found in our RISs analysis. To confirm the integration site near *MDS1* gene, we performed PCR with insertion specific primers amplifying the region between the 3′-LTR of the provirus and the *MDS1* locus. Furthermore, we used Q-PCR with Taqman probe to assess the level of clonal contribution to hematopoiesis from clones with *MDS1* RIS. The *MDS1* integration was not detected in the early phase, but became detectable at all time point from both lymphoid and myeloid populations from 6 months to 3 years post transplant (Figure 4).

We found a total of 15 common integration sites (CISs, as defined by Suzuki et al [14], Supplemental Table S4): 8 from the early and 10 from late samples, with 3 seen at both early and late phase. One CIS, *SEMA3E*, encodes a neuronal development protein which was recently found to be expressed on tumor cells [15]. No other CISs were near or within known oncogenes. Only 14.9% (20 of 134 early RISs) were CISs, while 36.8% (32 of 87 late RISs) were CISs (*p*-value 0.0002, Chi-square test).

## Discussion

Recent studies have demonstrated a bias of gammaretroviral vectors to integrate near TSSs [8], [16], [17] and those with strong enhancers have the ability to activate genes up to 100 Kb away [11]. Though our observed integration pattern is consistent with previous reports [18]–[20], the distribution of RISs among chromosomes assessed for both lymphoid and myeloid lineages appeared to differ between the early and late samples with a distinct pattern among lymphoid cells early post transplantation. Furthermore, only a small percentage (3.76%) of RISs was common among early and late samples. We have previously demonstrated in a large animal model that the initial clones detected early after transplant contribute only briefly, with clones derived from LT-HSCs detected at later time points [6], [21]. This low percentage of overlap offers further evidence that the early phase of hematopoiesis after transplantation derives primarily from a much greater number of short-term repopulating cells. This observation is consistent with other studies in which parallel transplantation of genetically modified CD34+ cells in both the baboon model and the immunodeficient mouse xenograft model demonstrated that only short term repopulating cells are read out in the xenograft model [22], [23]. A weakness of their conclusion, however, rests in the fact that only short term (12 weeks) time-points were analyzed in the xenograft model. Indeed, our results suggest that these early time-points would be predominantly derived from short term repopulating cells.

To ensure that the lack of overlap between samples did not reflect a sampling artifact and to circumvent the frequent nonsense sequences obtained using the TasI enzymes, TaiI enzymes were used. After optimization, over 1000 sequences were analyzed from which 213 unique integrations were obtained. Among the final 300 sequences obtained, no new RISs were identified, indicating there was adequate integration sampling. Our number of RISs is consistent with the 496 RISs reported from 5 ADA-SCID patients [19], 704 RISs reported from 9 SCID-X1 patients [20], and 439 RISs from 5 additional SCID-X1 patients [18].

We found total of 15 common integration sites (CISs) (8 from the early phase, 10 from the late phase, and 3 from both early and late phase). None of the CISs were near or within known oncogenes. Only 14.9% (20 of 134 early RISs) were CISs, while 36.8% (32 of 87 late RISs) were CISs (*p* = 0.0002). As the frequency of CISs increased in late phase populations, these results suggest that integrations at CISs dominate gene-modified long-term hematopoiesis. These observations after long term follow-up in a human gene therapy trial are in agreement with an emerging theory that vector integrations may serve as a tool to query genes involved in hematopoiesis in vivo [4], [24], [25].

This patient received 6.62×10^8^ transduced CD34+ cells, which theoretically contains approximately 1324 repopulating hematopoietic stem cells as the estimated frequency of LT-HSC within CD34+ populations is only 5 per 10^5^ cells [21], [26]. The overall gene marking level in this patient is 10^−3^ to 10^−2^% with an *MDS1* marking level of 10^−5^% in the follow-up samples, so the frequency of HSCs integrated at *MDS1* is estimated to be 1 per 1000. Even if the transduction efficiency of LT-HSC is equal to that of CD34+ cells (which was estimated 80–90% [27]) in this patient, then only one engrafted LT-HSC cell would be expected to have an integration at the *MDS1* locus. Regarding the risk of oncogenesis, it has been previously proposed that one must consider, as for any toxicology study, cell dose with the hypothesis being that limiting cell dose will limit side effects [10]. However, limiting numbers of gene marked LT-HSCs could paradoxically increase the risk of clonal dominance as such dominance appears more likely in the context of limited stem cell dose [26]. We infused an estimated 1324 transduced LT-HSC and an equivalent number of non-transduced LT-HSC. In this context of non-HSC limited, non-diseased hematopoiesis, the *MDS1* integrated LT-HSC would be predicted to have an insufficient advantage to become dominant.

In contrast, the clonal dominance of *MDS1* integrated cell populations observed in a recent chronic granulomatous disease (CGD) gene-therapy clinical trials could in part be explained by a lower infused cell dose along with the disease context [4]. We infused 4 times greater CD34 cells than that infused in the CGD trial. Further, half of them were non-transduced, and a significantly greater fraction of LT-HSCs were negative for the therapeutic and control vectors long term. These results argue that the integration around the *MDS1* region occurred in a LT-HSC and though theoretically it might impact engraftment or survival of the LT-HSC, it did not result in an abnormal proliferation, clonal expansion, or oncogenesis in our patient. Recent reports [28], [29] further characterized patients in whom insertional mutagenesis was observed: their analysis of integration sites and chromosomal rearrangements support the multi-hit leukemogenesis theory. Integration into oncogenes may be the initiating event, but oncogenesis requires additional upregulation of downstream transcription factors or the loss of tumor suppressor function. Additionally, previously described clonally expanded cells observed in human clinical trials to date occurred in the context of either a congenital immune-deficiency syndrome in which a selective advantage is known, or in the context of an LTR with known efficient expression among such expanded cells. These disease contexts thus may impose a skewed interpretation of in vivo hematopoiesis derived from retrovirally gene modified cells. Though the low levels of circulating genetically modified cells observed in our trial prevent a definitive interpretation, the absence of clonal outgrowth during extended follow up suggests that our integrations better reflect normal in vivo hematopoiesis.

In summary, the retroviral integration pattern observed in our HIV gene therapy trial is similar to that previously observed in model systems and human clinical trials, yet several novel observations warrant emphasis. The pattern of contribution by genetically modified cells is distinct between the early and late phase post transplantation and emphasizes the importance of long-term studies to assess the risk of integrating vectors. Additionally, the enrichment for CISs in the late phase supports the concept that integrations in the LT-HSCs favors genes that may be involved in “stemness” [24]. Furthermore, integrations in or near putative oncogenes are likely one step in the multi-hit process of oncogenesis. Finally, LT-HSC dose may be an important determinant of the risk of integrating vectors in the context of HSC gene transfer.

## Methods

The protocol was approved by the Institutional Scientific Review Committee and the Institutional Review Board of the National Heart, Lung, and Blood Institute, the Recombinant DNA Advisory Committee, and the Food and Drug Administration. All the study subjects gave written informed consent. The study design and outcome have been previously published [27], [30], registered at www.clinicaltrials.gov (NCT 00005785), and are described here briefly. An HIV positive patient with treatment-related AML underwent nonmyeloablative allogeneic transplantation from an HLA-matched sibling. Half of the donor cells were genetically modified with a Moloney murine leukemia virus (MoMLV) based HIV resistance vector containing a transdominant negative mutant Rev (TdRev) [31], [32] (2.58×10^8^ cells) or a control vector MoMLV based vector encoding gp91*phox* (4.04×10^8^ cells). The transduction efficiency was estimated by PCR, with 80% efficiency achieved from the TdRev aliquot and 90% for the gp91*phox* aliquot [27], [30]. The patient remained in complete remission from AML post transplant. Viral load remained undetectable and CD4 counts rose to over 500/mm^3^ long-term while continuing on the same highly active antiretroviral therapy (HAART) regimen until her death from thrombotic thrombocytopenic purpura 3 years and 4 months post transplant. Vector-transduced cells remained detectable at low levels. DNA was isolated from patient blood sample as previously described [33]. Peripheral blood granulocytes and lymphocytes were immunomagnetically selected as previously described on the basis of CD14/15 and CD3 expression, respectively. Marking levels were measured by Real-time PCR vector specific primers and overall marking was between 0.1% and 0.01% for both gp91*phox* and TdRev vectors in both lymphoid and myeloid cells at extended follow up [27], [30].

To identify genomic-proviral integration sites, LAM–PCR was performed as previously described [6]–[8] using primers listed in Supplemental Table S1 on DNA from blood samples obtained from one to three months (early) and 12 to 36 months (late) post-transplant as previously described [27], [30]. We used 2 different enzymes and designed 2 sets of LAM-PCR primers in order to maximize the number of integration sites amplified. First, TasI or TaiI enzymes (Fermantas, Hanover, MD) were used to digest genomic DNA followed by subsequent ligation to an asymmetric oligonucleotide linker cassette. Secondly, nested PCR was performed at two positions on the LTR to minimize primer interference. Each nested PCR primer was amplified for 32 reaction cycles. Junctions between 3′ long terminal repeats (LTRs) and genomic regions were separated and purified from 2.5% agarose gels and finally cloned with TOPO TA kit (Invitrogen, Carlsbad, CA). The criteria for verifying RISs are the sequence containing the remaining LTR sequence to junction points, the linker cassette sequence and sequence score of >90% identity to the human genome (UCSC BLAT to the May 2006 human genome assembly).

### Identification and Quantitation of RISs near MDS1/EVI1 gene by Real-time PCR analysis

To confirm the presence of an insertion near *MDS1*, PCR was performed on 10 ng DNA using the primer set in Table S1 in 30 cycles of amplification at 95° for 30 seconds, 55° for 30 seconds, and 72° for 1 minute. Quantification of the *MDS1* integrated clone's contributions over time after engraftment was determined by Real-time PCR analysis (QPCR) with Taqman probes as previously described [30]. QPCR was carried out on a Mx3000P®QPCR system (Stratagene, CA, USA) in a reaction mix containing one genomic primer, one vector primer, probes spanning the LTR-genomic junction, and Brilliant® II Q-PCR Master Mix (Stratagene) according to the manufacturer's instructions. Primers and probes were designed by using Real Time Design software (Biosearch Technology, CA, USA), Supplemental Table S1. β-actin gene number was used as a comparative control. The Mx3000P®QPCR system ran 50 cycles of amplification at 95°C for 25 seconds and 58°C for 60 seconds.

## Supporting Information

Table S1 (0.04 MB DOC)

Table S2 (0.13 MB DOC)

Table S3 (0.09 MB DOC)

Table S4 (0.04 MB DOC)
