# Supplementary material for: Long-Term Vector Integration Site Analysis Following Retroviral Mediated Gene Transfer to Hematopoietic Stem Cells for the Treatment of HIV Infection
Source: PLoS One. 2009 Jan 16;4(1):e4211. doi: 10.1371/journal.pone.0004211 (PMC2615408; doi:10.1371/journal.pone.0004211)
Supplement: Table S1 — (0.04 MB DOC) [file pone.0004211.s001.doc]

Hayakawa J et al.

**Long-term vector integration site analysis following retroviral mediated gene transfer to hematopoietic stem cells for the treatment of HIV infection**

Supplemental Table S1. List of primers used in PCR to analyze myeloid and lymphoid blood samples in our patient after allogeneic stem cell transplant.

|  | Primer | Sequence |
| --- | --- | --- |
| LAM-PCR | Linear amplification LTRb | 5'biotin-TTCCTG ACCTTGATCTGAACTTCTC-3' |
| Primer set 1 | LTR1 | 5'-AGCTTGCCTTGAGTGCTTCA-3’ |
|  | LC1 | 5'-TTCCTGACCTTGATCTGAACTTCTC-3’ |
|  | LTR2 | 5'-GCCTTGCAAAATGGCGTTAC-3’ |
|  | LC2 | 5'-GTGCGAGTAGCATACTAGAG-3’ |
| LAM-PCR |  |  |
| Primer set 2 | Linear amplification LTRb | 5'biotin-CTGACCTTGATCTGAACTTCTCTATTC-3' |
|  | LTR1 | 5'-TGTATTTTTCCATGCCTTGC-3’ |
|  | LTR2 | 5'-CCTTGCAAAATGGCGTTACT-3’ |
|  |  |  |
| Linker cassette | TasI | 5'-ACTGACAGCGGAGATAATCGGTGCGAGTAGCATACTAGAG-3' |
|  |  | 5'-AATTCTCTAGTATGCTACTCGCACCGATTATCTCCGCTGTCAGT-3' |
|  | TaiI | 5'-ACTGACAGCGGAGATAATCGGTGCGAGTAGCATACTAGAG-3' |
|  |  | 5'-TGCACTCTAGTATGCTACTCGCACCGATTATCTCCGCTGTCAGT-3' |
|  |  |  |
| PCR |  |  |
| vector specific primer1 | Nest1 Forward | 5'-GCCTTGCAAAATGGCGTTAC-3’ |
| vector specific primer2 | Nest2 Forward | 5'-GCTAGCTTG CCAAACCTACAG-3’ |
| MDS1 genomic primer1 | Nest1 Reverse | 5'-GTTTCCGACACCTCCCTTTC-3’ |
| MDS1 genomic primer2 | Nest2 Reverse | 5'-CAGGACCCGTACTCAAACC-3’ |
|  |  |  |
| Q-PCR (MDS1) | Forward | 5'-GCTTGCCAAACCTACAGGTG-3' |
|  | Reverse | 5'-TGGTGTCTGGCAGGATTTGC-3' |
|  | Tag man probe | 5'-TCACTTCCATGTGCTATTTATGTCCCCA-3' |
|  |  |  |
| Q-PCR (β-actin) | Forward | 5'-CATTGTGATGGACTCCGGAGACGG-3' |
|  | Reverse | 5'-CATCTCCTGCTCGAACTCTAGAGC-3' |
