# Supplementary material for: Long-Term Vector Integration Site Analysis Following Retroviral Mediated Gene Transfer to Hematopoietic Stem Cells for the Treatment of HIV Infection
Source: PLoS One. 2009 Jan 16;4(1):e4211. doi: 10.1371/journal.pone.0004211 (PMC2615408; doi:10.1371/journal.pone.0004211)
Supplement: Table S2 — (0.13 MB DOC) [file pone.0004211.s002.doc]

Hayakawa J et al.

**Long-term vector integration site analysis following retroviral mediated gene transfer to hematopoietic stem cells for the treatment of HIV infection**

Supplemental Table S2. List of retroviral integration sites in early (<3 months) myeloid and lymphoid blood samples in our patient after allogeneic stem cell transplant.

| **Number** | **Chromosome** | **Locus** | **Gene name** |
| --- | --- | --- | --- |
| 1 | 1 | (p36.11) | CNR2 (cannabinoid receptor 2) |
| 2 | 1 | (p36.11) | RPS6KA1(ribosomal protein S6 kinase, 90kDa, polypeptide) |
| 3 | 1 | (p34.3) | MACF1(microfilament and actin filament cross-linker) |
| 4 | 1 | (p34.2) | CAP1(adenylyl cyclase-associated protein) |
| 5 | 1 | (p34.2) | EBNA1BP2 (EBNA1 binding protein 2 ) |
| 6 | 1 | (p21.3) | DPYD(dihydropyrimidine dehydrogenase) |
| 7 | 1 | (p21.3) | SNX7 |
| 8 | 1 | (p21.3) | SNX7(sorting nexin 7 isoform a) |
| 9 | 1 | (q21.3) | AK125884(FLJ43896) |
| 10 | 1 | (q22) | ASH1L(ash1 (absent, small, or homeotic)-like) |
| 11 | 1 | (q25.2) | RASAL2(RAS protein activator like 2 isoform 2) |
| 12 | 1 | (q25.3) | ACBD6 (acyl-Coenzyme A binding domain containing 6 ) |
| 13 | 1 | (q32.1) | PLEKHA6(phosphoinositol 3-phosphate-binding protein-3) |
| 14 | 1 | (q42.12) |  |
| 15 | 2 | (p21) | ZFP36L2 (butyrate response factor 2) |
| 16 | 2 | (p15) | USP34 (ubiquitin specific protease 34 ) |
| 17 | 2 | (q12.3) |  |
| 18 | 2 | (q14.3) | CNTNAP5(contactin associated protein-like 5 isoform 1) |
| 19 | 2 | (q24.1) | GDP2(glycerol-3-phosphate dehydrogenase 2 ) |
| 20 | 2 | (q24.3) | FLJ39822(hypothetical protein LOC151258) |
| 21 | 2 | (q31.2) | SESTD1(SEC14 and spectrin domains 1) |
| 22 | 3 | (p24.1) | LRRC3B |
| 23 | 3 | (p24.1) | LRRC3B (leucine rich repeat containing 3B ) |
| 24 | 3 | (p24.1) | NEK10 (NIMA (never in mitosis gene a)- related kinase ) |
| 25 | 3 | (p22.2) | ITGA9 (integrin, alpha 9 precursor) |
| 26 | 3 | (p21.1) | CACNA1D (calcium channel, voltage-dependent, L type,) |
| 27 | 3 | (p21.1) | CACNA1D (calcium channel, voltage-dependent, L type,) |
| 28 | 3 | (p21.1) | CACNA1D (calcium channel, voltage-dependent, L type,) |
| 29 | 3 | (p21.1) | CACNA1D (calcium channel, voltage-dependent, L type,) |
| 30 | 3 | (q13.12) | BC101231(FLJ46100 ) |
| 31 | 3 | (q13.2) | CD96 (CD96 antigen isoform 1 precursor ) |
| 32 | 3 | (q13.2) | C3orf17(hypothetical protein LOC25871 isoform a) |
| 33 | 3 | (q21.1) | SEMA5B( semaphorin 5B isoform 1) |
| 34 | 3 | (q25.2) | BC038431 |
| 35 | 3 | (q26.33) |  |
| 36 | 4 | (p16.2) | ZNF509 (inc finger protein 509 ) |
| 37 | 4 | (p15.31) | KCNIP4(Kv channel interacting protein 4) |
| 38 | 4 | (q13.1) | LPHN3(: latrophilin 3 precursor) |
| 39 | 4 | (q13.3) | SLC4A4 (solute carrier family 4, sodium bicarbonate ) |
| 40 | 4 | (q21.3) | PTPN13(protein tyrosine phosphatase, non-receptor type) |
| 41 | 4 | (q32.1) | NPY2R(neuropeptide Y receptorY2) |
| 42 | 4 | (q32.1) | GRIA2(glutamate receptor, ionotropic, AMPA 2) |
| 43 | 4 | (q34.3) |  |
| 44 | 5 | (q14.3) |  |
| 45 | 5 | (q21.1) |  |
| 46 | 5 | (q31.1) | FNIP1(folliculin interacting protein 1 isoform 1) |
| 47 | 5 | (q33.1) | FAT2(FAT tumor suppressor 2 precursor) |
| 48 | 6 | (p25.2) | LOC401233 |
| 49 | 6 | (p23) |  |
| 50 | 6 | (q15) | ANKRD6 (ankyrin repeat domain 6 ) |
| 51 | 6 | (q21) | SLC22A16(solute carrier family 22, member 16) |
| 52 | 7 | (p22.3) | INTS1 (FLJ36490) |
| 53 | 7 | (p22.1) | AK123300(FLJ41306) |
| 54 | 7 | (p22.1) | AK123300(FLJ41306) |
| 55 | 7 | (q33.1) |  |
| 56 | 7 | (p21.1) |  |
| 57 | 7 | (q11.23) | LOC554248 |
| 58 | 7 | (q31.1) |  |
| 59 | 7 | (q32.1) | IRF5 (interferon regulatory factor 5 isoform a) |
| 60 | 8 | (q13.2) | DEPDC2 (DEP domain containing 2 isoform a ) |
| 61 | 8 | (q24.13) |  |
| 62 | 9 | (q21.13) | ALDH1A1 (aldehyde dehydrogenase 1A1) |
| 63 | 9 | (q21.13) | VPS13A (vacuolar protein sorting 13A isoform D ) |
| 64 | 9 | (q22.2) |  |
| 65 | 9 | (q34.11) | FNBP1(ormin binding protein 1) |
| 66 | 9 | (q34.11) | FNBP1 (formin binding protein 1) |
| 67 | 9 | (q34.11) | FNBP1 (formin binding protein 1) |
| 68 | 9 | (q34.11) | FNBP1 (formin binding protein 1) |
| 69 | 10 | (q11.22) | L25628 |
| 70 | 10 | (q11.22) |  |
| 71 | 10 | (q21.1) |  |
| 72 | 10 | (q23.2) | PAPSS2 (3'-phosphoadenosine 5'-phosphosulfate synthase 2) |
| 73 | 10 | (q24.32) | C10orf76(hypothetical protein LOC79591) |
| 74 | 11 | (p15.5) | Uncharacterized protein C11orf21. |
| 75 | 11 | (p15.4) | DCHS1(dachsous 1 precursor) |
| 76 | 11 | (p15.4) | SWAP70 |
| 77 | 11 | (p15.4) | XLKD1 (extracellular link domain containing 1 ) |
| 78 | 11 | (p14.3) | LUZP2 (leucine zipper protein 2 ) |
| 79 | 11 | (p13) | CCDC73 (sarcoma antigen NY-SAR-79) |
| 80 | 11 | (p13) | LDLRAD3( low density lipoprotein receptor class A domain) |
| 81 | 11 | (q12.1) | LPXN(leupaxin) |
| 82 | 11 | (q13.2) | CPT1A(carnitine palmitoyltransferase 1A isoform 2） |
| 83 | 11 | (q23.1) | IL-18 |
| 84 | 11 | (q24.1) | SORL1 (sortilin-related receptor containing LDLR class) |
| 85 | 12 | (p13.2) | STYK1(serine/threonine/tyrosine kinase 1) |
| 86 | 12 | (q13.11) | HDAC7A(histone deacetylase 7A isoform a) |
| 87 | 12 | (q14.1) |  |
| 88 | 12 | (q23.1) | ELK3 protein |
| 89 | 12 | (q23.2) | C12orf48（hypothetical protein LOC55010） |
| 90 | 12 | (q24.11) | SVOP(SV2 related protein) |
| 91 | 13 | (q12.11) | GJB6 ( gap junction protein, beta 6 ) |
| 92 | 13 | (q12.11) | CRYL1( lambda-crystallin) |
| 93 | 13 | (q14.3) | ARL11(ADP-ribosylation factor-like 11) |
| 94 | 13 | (q21.1) |  |
| 95 | 13 | (q21.31) |  |
| 96 | 13 | (q22.1) | LOC440145 |
| 97 | 14 | (q11.2) | TCRA(Homo sapiens T cell recptor alpha chain (TCRA) mRNA) |
| 98 | 14 | (q13.3) | MIPOL1（mirror-image polydactyly 1） |
| 99 | 14 | (q32.33) |  |
| 100 | 15 | (q11.2) |  |
| 101 | 15 | (q21.3) |  |
| 102 | 15 | (q21.3) |  |
| 103 | 15 | (q21.3) |  |
| 104 | 16 | (p13.3) | BC108660 (Homo sapiens cDNA clone IMAGE:5244947 ) |
| 105 | 16 | (p12.1) | UQCRC2(ubiquinol-cytochrome c reductase core protein) |
| 106 | 16 | (p12.1) | FLJ36063 |
| 107 | 16 | (q12.2) | CHD9(chromodomain helicase DNA binding protein 9) |
| 108 | 16 | (q22.1) | C16orf70 |
| 109 | 17 | (p13.3) | SERPINF1(serine (or cysteine) proteinase inhibitor) |
| 110 | 17 | (p13.1) | AURKB(aurora kinase B) |
| 111 | 17 | (p12) | ADORA2B(adenosine A2b receptor) |
| 112 | 17 | (q11.2) | SEZ6 (seizure related 6 homolog ) |
| 113 | 18 | (p11.31) | LPIN2(lipin 2) |
| 114 | 18 | (q12.2) |  |
| 115 | 18 | (q21.32) | ZNF532 (zinc finger protein 532) |
| 116 | 18 | (q22.3) | NETO1(neuropilin- and tolloid-like protein 1 isoform 2) |
| 117 | 19 | (p13.13) | CCDC130(coiled-coil domain containing 130) |
| 118 | 19 | (q13.11) | ZNF792(Homo sapiens cDNA FLJ38451) |
| 119 | 19 | (q13.42) | NLRP11(NACHT, leucine rich repeat and PYD containing) |
| 120 | 19 | (q13.42) | NLRP11(NACHT, leucine rich repeat and PYD containing) |
| 121 | 19 | (q13.42) | NLRP11(NACHT, leucine rich repeat and PYD containing) |
| 122 | 20 | (q11.22) | TTLL9(tubulin tyrosine ligase-like family, member 9) |
| 123 | 21 | (q22.3) | C21orf25(hypothetical protein LOC25966 isoform 1) |
| 124 | 21 | (q22.3) | ABCG1(ATP-binding cassette sub-family G member 1) |
| 125 | 21 | (q22.3) | ABCG1(ATP-binding cassette sub-family G member 1) |
| 126 | 21 | (q22.3) | PDE9A (phosphodiesterase 9A isoform a) |
| 127 | X | (p21.1) | DMD (dystrophin Dp427c isoform ) |
| 128 | X | (p11.4) | CYBB(cytochrome b-245, beta polypeptide) |
| 129 | X | (p11.4) |  |
| 130 | X | (p11.21) | ITIH5L(inter-alpha (globulin) inhibitor H5-like) |
| 131 | X | (q21.1) | POF1B(premature ovarian failure, 1B) |
| 132 | X | (q22.1) | SYTL4 (synaptotagmin-like 4 ) |
| 133 | X | (q22.1) | CENPI(Centromere protein I ) |
| 134 | X | (q22.3) | IL1RAPL2(interleukin 1 receptor accessory protein-like 2) |
