# Supplementary material for: Long-Term Vector Integration Site Analysis Following Retroviral Mediated Gene Transfer to Hematopoietic Stem Cells for the Treatment of HIV Infection
Source: PLoS One. 2009 Jan 16;4(1):e4211. doi: 10.1371/journal.pone.0004211 (PMC2615408; doi:10.1371/journal.pone.0004211)
Supplement: Table S3 — (0.09 MB DOC) [file pone.0004211.s003.doc]

Hayakawa J et al.

**Long-term vector integration site analysis following retroviral mediated gene transfer to hematopoietic stem cells for the treatment of HIV infection**

Supplemental Table S3. List of retroviral integration sites in late (>1 year) myeloid and lymphoid blood samples in our patient after allogeneic stem cell transplant.

| **Number** | **Chromosome** | **Locus** | **Gene name** |
| --- | --- | --- | --- |
| 1 | 1 | (q32.2) | sytXIV(sytXIV mRNA for synaptotagmin XIV) |
| 2 | 2 | (p16.1) |  |
| 3 | 2 | (p11.2) | FLJ10916(hypothetical protein LOC55258) |
| 4 | 2 | (q14.2) | LOC130355 |
| 5 | 2 | (q21.1) |  |
| 6 | 2 | (q24.3) | FLJ39822(hypothetical protein LOC151258) |
| 7 | 3 | (p24.1) | EOMES(eomesodermin) |
| 8 | 3 | (p22.1) |  |
| 9 | 3 | (p21.1) | CACNA1D( calcium channel, voltage-dependent, L type) |
| 10 | 3 | (p21.1) | CACNA1D( calcium channel, voltage-dependent, L type) |
| 11 | 3 | (p21.1) | CACNA1D( calcium channel, voltage-dependent, L type) |
| 12 | 3 | (p21.1) | CACNA1D( calcium channel, voltage-dependent, L type) |
| 13 | 3 | (q12.3) | NFKBIZ(nuclear factor of kappa light polypeptide gene ) |
| 14 | 3 | (q13.33) | LRRC58 |
| 15 | 3 | (q26.2) | MDS1(myelodysplasia syndrome protein 1) |
| 16 | 3 | (q27.1) | YEATS2 |
| 17 | 4 | (q12) | GSH2 homeobox protein GSH-2 |
| 18 | 4 | (q35.1) |  |
| 19 | 5 | (q14.3) | EDIL3 (EGF-like repeats and discoidin I-like ) |
| 20 | 5 | (q14.3) |  |
| 21 | 5 | (q15) |  |
| 22 | 5 | (q21.3) | AK093222 (FLJ35903 ) |
| 23 | 5 | (q23.2) | MARCH3(membrane-associated ring finger (C3HC4) ) |
| 24 | 5 | (q23.2) | MARCH3(membrane-associated ring finger (C3HC4) ) |
| 25 | 6 | (p25.1) | FARS( phenylalanyl-tRNA synthetase 2 precursor) |
| 26 | 6 | (p24.1) | HIVEP1( human immunodeficiency virus type I enhancer) |
| 27 | 6 | (p22.3) | ATXN1（ataxin 1） |
| 28 | 6 | (q12) |  |
| 29 | 6 | (q21） | WISP3 （WNT1-inducible-signaling pathway protein 3 precursor ） |
| 30 | 7 | (p22.1) | AK123300(FLJ41306) |
| 31 | 7 | (p22.1) | AK123300(FLJ41306) |
| 32 | 7 | (p22.1) | AK123300(FLJ41306) |
| 33 | 7 | (p22.1) | AK123300(FLJ41306) |
| 34 | 7 | (p22.1) | AK123300(FLJ41306) |
| 35 | 7 | (p14.1) |  |
| 36 | 7 | (q21.11) | SEMA3E(semaphorin 3E) |
| 37 | 7 | (q21.11) | SEMA3E(semaphorin 3E) |
| 38 | 7 | (q21.11) |  |
| 39 | 7 | (q21.13) | PFTK1 (PFTAIRE protein kinase 1) |
| 40 | 8 | (p23.1) | MSRA(methionine sulfoxide reductase A) |
| 41 | 8 | (p22) |  |
| 42 | 8 | (q21.11) |  |
| 43 | 9 | (q31.3) | OR2K2(olfactory receptor, family 2, subfamily K,) |
| 44 | 9 | (q32) | DFNB31(CASK-interacting protein CIP98) |
| 45 | 9 | (q34.11) | FNBP1(formin binding protein 1) |
| 46 | 9 | (q34.11) | FNBP1(formin binding protein 1) |
| 47 | 9 | (q34.11) | FNBP1(formin binding protein 1) |
| 48 | 9 | (q34.11) | FNBP1(formin binding protein 1) |
| 49 | 9 | (q34.11) | FNBP1(formin binding protein 1) |
| 50 | 9 | (q34.11) | FNBP1(formin binding protein 1) |
| 51 | 9 | (q34.11) | FNBP1(formin binding protein 1) |
| 52 | 9 | (q34.11) | FNBP1(formin binding protein 1) |
| 53 | 9 | (q34.11) | FNBP1(formin binding protein 1) |
| 54 | 10 | (q11.22) | CR604707 |
| 55 | 11 | (p15.4) | XLKD1 (extracellular link domain containing 1) |
| 56 | 11 | (p15.2) | TEAD1(TEA domain family member 1) |
| 57 | 11 | (q24.1) | SORL1 (sortilin-related receptor containing LDLR class) |
| 58 | 11 | (q24.1) | SORL1 (sortilin-related receptor containing LDLR class) |
| 59 | 12 | (p13.2) | CSDA(cold shock domain protein A ) |
| 60 | 12 | (p13.1) | C12orf36 (hypothetical protein LOC283422 ) |
| 61 | 12 | (q13.11) | HDAC7A(histone deacetylase 7A isoform a) |
| 62 | 12 | (q13.11) | HDAC7A(histone deacetylase 7A isoform a) |
| 63 | 12 | (q14.1) | FAM19A2(family with sequence similarity 19 (chemokine) |
| 64 | 12 | (q21.1) | LGR5 (leucine-rich repeat-containing G protein-coupled) |
| 65 | 12 | (q21.1) | LGR5 (leucine-rich repeat-containing G protein-coupled) |
| 66 | 12 | (q32.2) | PMCH( pro-melanin-concentrating hormone) |
| 67 | 13 | (q12.12) | FLJ00188 |
| 68 | 13 | (q21.1) |  |
| 69 | 13 | (q21.13) |  |
| 70 | 13 | (q22.2) | COMMD6(COMM domain containing 6 isoform a) |
| 71 | 14 | (q11.2) | TCRA(Homo sapiens T cell recptor alpha chain (TCRA) mRNA) |
| 72 | 14 | (q13.3) | MIPOL1（mirror-image polydactyly 1） |
| 73 | 14 | (q24.2) | RGS6( regulator of G-protein signalling 6) |
| 74 | 15 | (q13.3) | CHRNA7(cholinergic receptor, nicotinic, alpha 7) |
| 75 | 15 | (q21.3) |  |
| 76 | 15 | (q21.3) |  |
| 77 | 16 | (q21) |  |
| 78 | 17 | (p12) | ADORA2B( adenosine A2b receptor) |
| 79 | 17 | (p12) | ADORA2B( adenosine A2b receptor) |
| 80 | 17 | (q24.2) | ABCA8 (ATP-binding cassette, sub-family A member 8 ) |
| 81 | 18 | (p11.31) | LPIN2(lipin 2) |
| 82 | 18 | (p11.23) |  |
| 83 | 18 | (q21.31) | ATP8B1(ATPase, Class I, type 8B, member 1) |
| 84 | 20 | (q13.13) | ARFGEF2 ( ADP-ribosylation factor guanine ) |
| 85 | 21 | (q22.3) | ABCG1(ATP-binding cassette sub-family G member 1) |
| 86 | X | (p22.2) |  |
| 87 | X | (q22.1) | CENPI ( follicle-stimulating hormone primary response) |
