# Supplementary material for: Long-Term Vector Integration Site Analysis Following Retroviral Mediated Gene Transfer to Hematopoietic Stem Cells for the Treatment of HIV Infection
Source: PLoS One. 2009 Jan 16;4(1):e4211. doi: 10.1371/journal.pone.0004211 (PMC2615408; doi:10.1371/journal.pone.0004211)
Supplement: Table S4 — (0.04 MB DOC) [file pone.0004211.s004.doc]

Hayakawa J et al.

**Long-term vector integration site analysis following retroviral mediated gene transfer to hematopoietic stem cells for the treatment of HIV infection**

Supplemental Table S4. Common integration sites in early (<3 months) and late (>1 year) myeloid and lymphoid blood samples in our patient after allogeneic stem cell transplant.

| Gene name | Gene function | <3 month myeloid | <3 month lymphoid | >1 year myeloid | >1 year lymphoid |
| --- | --- | --- | --- | --- | --- |
| *SNX7* | Intracellular trafficking |  | Yes |  |  |
| *LRRC3B* | unknown |  | Yes |  |  |
| *CACNA1D* | Cell signaling, cell motility | Yes |  | Yes | yes |
| *AK123300* | unknown |  | Yes | Yes | Yes |
| *FNBP1* | Cytoskeleton regulation | Yes | Yes | Yes | yes |
| *q21.3* | Unknown | Yes | Yes | Yes | Yes |
| *NLRP11* | Pro-inflammatory, caspase expression | Yes | Yes |  |  |
| *ABCG1* | Cholesterol transport | Yes | Yes |  |  |
| *SORL1* | Neuronal apolipoprotein E receptor |  |  | Yes | Yes |
| *MARCH3* | Endosomal trafficking |  |  | Yes | Yes |
| *SEMA3E* | Neuronal cell development, expression on tumor cell lines |  |  | Yes | Yes |
| *HDAC7* | Chromatin structure |  |  | Yes | Yes |
| *LGR5* | Cell signaling |  |  | Yes |  |
| *ADORA2B* |  |  |  | Yes |  |
